# *N*-Cyclo­hexyl-5*H*,7*H*-13,15-dimethyl-9-nitro-5-oxophenanthrido[4,4a,5-*bc*][1,4]benzoxazepine-7-carboxamide

**DOI:** 10.1107/S1600536809028712

**Published:** 2009-07-25

**Authors:** Jia-Lu Luo, Jin-Long Wu

**Affiliations:** a Laboratory of Asymmetric Catalysis and Synthesis, Department of Chemistry, Zhejiang University, Hangzhou, Zhejiang 310027, People’s Republic of China

## Abstract

In the title compound, C_29_H_27_N_3_O_5_, a dibenz[*b*,*f*][1,4]oxazepine derivative, the cyclo­hexane ring adopts a chair conformation, the oxazepine seven-membered ring has a twist-boat conformation, and the piperidin-2-one ring assumes a flattened boat conformation. Inter­molecular N—H⋯O hydrogen bonding between imino and nitro groups links two mol­ecules into a centrosymmetric dimer.

## Related literature

For the biological activity of dibenz[*b*,*f*][1,4]oxazepines, see: Klunder *et al.* (1992 ▶); Merluzzi *et al.* (1990 ▶); Nagarajan *et al.* (1986 ▶); Hallinan *et al.* (1993 ▶, 1996 ▶). For our recent microwave-assisted synthesis of dibenz[*b*,*f*][1,4]oxazepines, see: Dai & Shi (2007 ▶); Xing *et al.* (2006 ▶). For microwave-assisted palladium-catalysed intra­molecular direct aryl­ation, see: Wu *et al.* (2007 ▶).
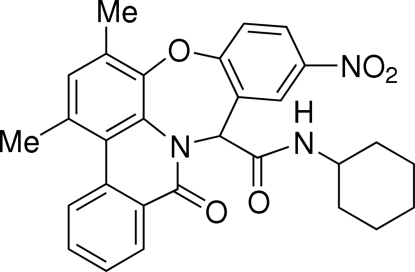


## Experimental

### 

#### Crystal data

C_29_H_27_N_3_O_5_
*M* *_r_* = 497.54
Monoclinic, 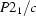

*a* = 10.7451 (4) Å
*b* = 27.8791 (7) Å
*c* = 8.4917 (3) Å
β = 105.428 (13)°
*V* = 2452.1 (2) Å^3^
*Z* = 4
Mo *K*α radiation
μ = 0.09 mm^−1^
*T* = 296 K
0.28 × 0.26 × 0.11 mm

#### Data collection

Rigaku R-AXIS RAPID diffractometer
Absorption correction: none
23870 measured reflections
5594 independent reflections
3756 reflections with *I* > 2σ(*I*)
*R*_int_ = 0.054

#### Refinement

*R*[*F* ^2^ > 2σ(*F* ^2^)] = 0.051
*wR*(*F* ^2^) = 0.127
*S* = 1.00
5594 reflections
336 parameters
H-atom parameters constrained
Δρ_max_ = 0.29 e Å^−3^
Δρ_min_ = −0.23 e Å^−3^

### 

Data collection: *PROCESS-AUTO* (Rigaku, 2006 ▶); cell refinement: *PROCESS-AUTO*; data reduction: *CrystalStructure* (Rigaku, 2007 ▶); program(s) used to solve structure: *SIR97* (Altomare *et al.*, 1999 ▶); program(s) used to refine structure: *SHELXL97* (Sheldrick, 2008 ▶); molecular graphics: *ORTEP-3 for Windows* (Farrugia, 1997 ▶); software used to prepare material for publication: *CrystalStructure*.

## Supplementary Material

Crystal structure: contains datablocks global, I. DOI: 10.1107/S1600536809028712/xu2558sup1.cif

Structure factors: contains datablocks I. DOI: 10.1107/S1600536809028712/xu2558Isup2.hkl

Additional supplementary materials:  crystallographic information; 3D view; checkCIF report

## Figures and Tables

**Table 1:** Hydrogen-bond geometry (Å, °)

*D*—H⋯*A*	*D*—H	H⋯*A*	*D*⋯*A*	*D*—H⋯*A*
N3—H3⋯O3^i^	0.86	2.29	3.046 (2)	147

Symmetry code: (i) 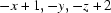.

**Fig. 1.**
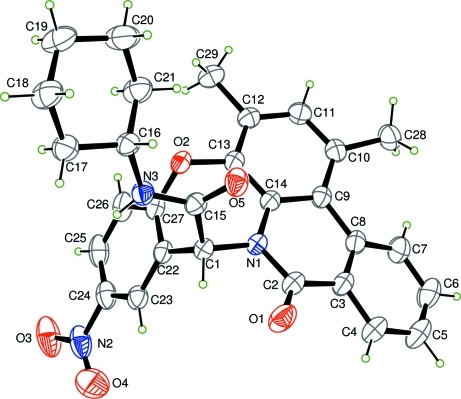
A view of (1). Displacement ellipsoids are drawn at 40% probability level and H atoms are shown as small circles of arbitary radii.

## supplementary crystallographic information

### Comment

The title compound, C_29_H_27_N_3_O_5_, is viewed as the derivative of dibenz[*b*,*f*][1,4]oxazepines, which have been reported to deliver various biological activities such as non-nucleoside inhibitor of HIV-1 reverse transcriptase (Klunder *et al.*, 1992; Merluzzi *et al.*, 1990), antidepressant (Nagarajan *et al.*, 1986), and PGE_2_ antagonist and analgesic (Hallinan *et al.*, 1993, 1996). The title compound has recently been obtained during microwave-assisted synthesis of a derivative of dibenz[*b*,*f*][1,4]oxazepines (Dai & Shi, 2007; Xing *et al.*, 2006) with a microwave-assisted palladium-catalyzed intramolecular direct arylation reaction (Wu *et al.*, 2007). We report here its crystal structure.

In the molecular structure (Fig. 1) there are one cyclohexane, one oxazepine and one piperidin-2-one rings. The cyclohexane ring adopts a chair conformation with atoms C16 and C19 deviated from the mean plane formed by the other four atoms by 0.677 (3) and -0.673 (4) Å, respectively. The 7-membered oxazepine ring has a twist-boat conformation, and the piperidin-2-one assumes a flatboat conformation. Intermolecular N—H···O hydrogen-bond bonding between imino and nitro groups (Table 1) links two molecules into the centro-symmetric dimer.

### Experimental

A 10-ml pressurized process vial was charged with the bromide (59.5 mg, 0.10 mmol), Pd(OAc)_2_ (0.6 mg, 0.0026 mmol), 1,1'-bis(diphenylphosphino)ferrocene (1.6 mg, 0.0028 mmol), and K_2_CO_3_ (27.7 mg, 0.20 mmol) and it was sealed with a cap containing a silicon septum. The vial was then evacuated and backfilled with N_2_ (repeated for several times) through the cap using a needle. To the degassed vial was added degassed anhydrous PhMe (2 ml) through the cap using a syringe. The loaded vial was then placed into the microwave reactor cavity and was heated at 423 K for 1 h. After cooled to room temperature the resultant mixture was filtered off through a plug of Celite with washing by EtOAc. The combined filtrate was evaporated under reduced pressure. The residue was purified by column chromatography (silica gel, 20% EtOAc in petroleum ether) to furnish the title compound in 95% yield (48.5 mg) as a pale yellow solid. m.p. > 555 K (EtOAc-hexane). Single crystals suitable for X-ray diffraction of the title compound were grown in the mixed solvent of ethyl acetate and hexane.

### Refinement

All H atoms were placed in calculated positions with C—H = 0.93–0.98Å and N—H = 0.86 Å and included in the refinement in riding model, with *U*_iso_(H) = 1.2 *U*_eq_(C,N). The H atoms of one methyl group are equally disordered over two sites.

### Crystal data

**Table:**

C_29_H_27_N_3_O_5_	*F*(000) = 1048
*M**_r_* = 497.54	*D*_x_ = 1.348 Mg m^−^^3^
Monoclinic, *P*2_1_/*c*	Mo *K*α radiation, λ = 0.71073 Å
Hall symbol: -P 2ybc	Cell parameters from 15928 reflections
*a* = 10.7451 (4) Å	θ = 3.1–27.5°
*b* = 27.8791 (7) Å	µ = 0.09 mm^−^^1^
*c* = 8.4917 (3) Å	*T* = 296 K
β = 105.428 (13)°	Chunk, yellow
*V* = 2452.1 (2) Å^3^	0.28 × 0.26 × 0.11 mm
*Z* = 4	

### Data collection

**Table:**

Rigaku R-AXIS RAPID diffractometer	3756 reflections with *I* > 2σ(*I*)
Radiation source: rolling anode	*R*_int_ = 0.054
graphite	θ_max_ = 27.5°, θ_min_ = 3.1°
Detector resolution: 10.00 pixels mm^-1^	*h* = −13→13
ω scans	*k* = −36→35
23870 measured reflections	*l* = −10→10
5594 independent reflections	

### Refinement

**Table:**

Refinement on *F*^2^	Secondary atom site location: difference Fourier map
Least-squares matrix: full	Hydrogen site location: inferred from neighbouring sites
*R*[*F*^2^ > 2σ(*F*^2^)] = 0.051	H-atom parameters constrained
*wR*(*F*^2^) = 0.127	*w* = 1/[σ^2^(*F*_o_^2^) + (0.0432*P*)^2^ + 1*P*] where *P* = (*F*_o_^2^ + 2*F*_c_^2^)/3
*S* = 1.00	(Δ/σ)_max_ < 0.001
5594 reflections	Δρ_max_ = 0.29 e Å^−^^3^
336 parameters	Δρ_min_ = −0.23 e Å^−^^3^
0 restraints	Extinction correction: *SHELXL97* (Sheldrick, 2008), Fc^*^=kFc[1+0.001xFc^2^λ^3^/sin(2θ)]^-1/4^
Primary atom site location: structure-invariant direct methods	Extinction coefficient: 0.0051 (10)

### Special details

**Table:**

Geometry. All e.s.d.'s (except the e.s.d. in the dihedral angle between two l.s. planes) are estimated using the full covariance matrix. The cell e.s.d.'s are taken into account individually in the estimation of e.s.d.'s in distances, angles and torsion angles; correlations between e.s.d.'s in cell parameters are only used when they are defined by crystal symmetry. An approximate (isotropic) treatment of cell e.s.d.'s is used for estimating e.s.d.'s involving l.s. planes.
Refinement. Refinement of *F*^2^ against ALL reflections. The weighted *R*-factor *wR* and goodness of fit *S* are based on *F*^2^, conventional *R*-factors *R* are based on *F*, with *F* set to zero for negative *F*^2^. The threshold expression of *F*^2^ > σ(*F*^2^) is used only for calculating *R*-factors(gt) *etc*. and is not relevant to the choice of reflections for refinement. *R*-factors based on *F*^2^ are statistically about twice as large as those based on *F*, and *R*- factors based on ALL data will be even larger.

### Fractional atomic coordinates and isotropic or equivalent isotropic displacement parameters (Å^2^)

**Table:**

	*x*	*y*	*z*	*U*_iso_*/*U*_eq_	Occ. (<1)
O2	0.52077 (12)	0.14876 (4)	0.75758 (15)	0.0441 (3)	
N1	0.30895 (13)	0.12575 (5)	0.46737 (17)	0.0363 (3)	
O5	0.53052 (13)	0.12552 (5)	0.37217 (15)	0.0509 (4)	
C13	0.43278 (17)	0.18313 (6)	0.6721 (2)	0.0371 (4)	
C14	0.32745 (16)	0.17213 (6)	0.5375 (2)	0.0355 (4)	
N3	0.63285 (14)	0.07287 (6)	0.56536 (19)	0.0429 (4)	
H3	0.6229	0.0498	0.6281	0.052*	
C10	0.27335 (18)	0.25731 (7)	0.5195 (2)	0.0423 (4)	
C9	0.23969 (17)	0.20945 (6)	0.4689 (2)	0.0383 (4)	
C22	0.40912 (16)	0.07561 (6)	0.7002 (2)	0.0381 (4)	
O1	0.20934 (15)	0.07613 (5)	0.25999 (19)	0.0654 (4)	
C12	0.46090 (18)	0.22939 (7)	0.7269 (2)	0.0419 (4)	
C1	0.40183 (16)	0.08658 (6)	0.5246 (2)	0.0364 (4)	
H1	0.3657	0.0581	0.4615	0.044*	
C16	0.76257 (17)	0.08427 (6)	0.5510 (2)	0.0406 (4)	
H16	0.7586	0.0869	0.4347	0.049*	
C3	0.10767 (18)	0.15002 (7)	0.2797 (2)	0.0437 (4)	
C11	0.38132 (19)	0.26588 (7)	0.6456 (2)	0.0453 (4)	
H11	0.4019	0.2974	0.6779	0.054*	
C27	0.46631 (17)	0.10965 (7)	0.8143 (2)	0.0411 (4)	
C15	0.52969 (17)	0.09773 (6)	0.4828 (2)	0.0378 (4)	
C2	0.21089 (18)	0.11460 (7)	0.3296 (2)	0.0440 (4)	
N2	0.3006 (2)	−0.01158 (8)	0.9665 (3)	0.0698 (6)	
C8	0.11937 (18)	0.19597 (7)	0.3490 (2)	0.0430 (4)	
C23	0.35448 (18)	0.03542 (7)	0.7497 (2)	0.0455 (5)	
H23	0.3150	0.0120	0.6749	0.055*	
C24	0.3602 (2)	0.03099 (8)	0.9136 (3)	0.0521 (5)	
C26	0.4739 (2)	0.10462 (8)	0.9787 (2)	0.0531 (5)	
H26	0.5151	0.1276	1.0540	0.064*	
O3	0.3241 (2)	−0.01856 (7)	1.1134 (2)	0.0931 (7)	
C25	0.4187 (2)	0.06458 (8)	1.0275 (3)	0.0581 (6)	
H25	0.4211	0.0604	1.1369	0.070*	
C4	−0.0059 (2)	0.13491 (9)	0.1673 (3)	0.0583 (6)	
H4	−0.0109	0.1043	0.1223	0.070*	
C28	0.2043 (2)	0.30111 (7)	0.4341 (3)	0.0576 (5)	
H28A	0.1288	0.3071	0.4712	0.086*	
H28B	0.1793	0.2957	0.3183	0.086*	
H28C	0.2609	0.3283	0.4585	0.086*	
C29	0.5782 (2)	0.24052 (8)	0.8644 (3)	0.0555 (5)	
H29A	0.5820	0.2744	0.8856	0.067*	0.50
H29B	0.6544	0.2307	0.8345	0.067*	0.50
H29C	0.5729	0.2236	0.9608	0.067*	0.50
H29D	0.6243	0.2114	0.9017	0.067*	0.50
H29E	0.5518	0.2551	0.9528	0.067*	0.50
H29F	0.6333	0.2622	0.8265	0.067*	0.50
C21	0.8092 (2)	0.13172 (8)	0.6321 (3)	0.0585 (6)	
H21A	0.8113	0.1302	0.7469	0.070*	
H21B	0.7498	0.1570	0.5819	0.070*	
C7	0.0090 (2)	0.22515 (8)	0.3054 (3)	0.0586 (6)	
H7	0.0109	0.2553	0.3529	0.070*	
C19	1.0384 (2)	0.10368 (9)	0.6890 (3)	0.0697 (7)	
H19A	1.1223	0.1111	0.6728	0.084*	
H19B	1.0475	0.1017	0.8055	0.084*	
C18	0.9917 (2)	0.05620 (8)	0.6100 (3)	0.0624 (6)	
H18A	1.0508	0.0311	0.6621	0.075*	
H18B	0.9910	0.0573	0.4956	0.075*	
O4	0.2348 (2)	−0.03785 (8)	0.8627 (3)	0.1074 (8)	
C17	0.85656 (19)	0.04450 (8)	0.6240 (3)	0.0560 (5)	
H17A	0.8275	0.0146	0.5676	0.067*	
H17B	0.8588	0.0403	0.7382	0.067*	
C5	−0.1100 (2)	0.16528 (10)	0.1235 (3)	0.0711 (7)	
H5	−0.1851	0.1557	0.0470	0.085*	
C6	−0.1020 (2)	0.21008 (10)	0.1942 (3)	0.0717 (7)	
H6	−0.1729	0.2305	0.1661	0.086*	
C20	0.9432 (2)	0.14320 (9)	0.6151 (4)	0.0747 (7)	
H20A	0.9397	0.1468	0.5004	0.090*	
H20B	0.9725	0.1733	0.6696	0.090*	

### Atomic displacement parameters (Å^2^)

**Table:**

	*U*^11^	*U*^22^	*U*^33^	*U*^12^	*U*^13^	*U*^23^
O2	0.0353 (7)	0.0476 (7)	0.0436 (7)	0.0026 (6)	0.0003 (6)	0.0016 (6)
N1	0.0318 (7)	0.0418 (8)	0.0331 (7)	0.0010 (6)	0.0050 (6)	−0.0007 (6)
O5	0.0476 (8)	0.0657 (9)	0.0409 (7)	0.0051 (7)	0.0144 (6)	0.0145 (7)
C13	0.0336 (9)	0.0430 (9)	0.0344 (9)	0.0014 (7)	0.0084 (7)	0.0016 (7)
C14	0.0333 (9)	0.0417 (9)	0.0324 (8)	−0.0011 (7)	0.0104 (7)	0.0008 (7)
N3	0.0347 (8)	0.0480 (9)	0.0470 (9)	0.0021 (7)	0.0125 (7)	0.0101 (7)
C10	0.0417 (10)	0.0443 (10)	0.0441 (10)	0.0042 (8)	0.0169 (9)	0.0052 (8)
C9	0.0356 (9)	0.0453 (10)	0.0349 (9)	0.0020 (8)	0.0111 (7)	0.0045 (7)
C22	0.0305 (9)	0.0470 (10)	0.0362 (9)	0.0065 (8)	0.0075 (7)	0.0064 (8)
O1	0.0584 (9)	0.0635 (9)	0.0603 (9)	0.0070 (8)	−0.0088 (7)	−0.0223 (8)
C12	0.0398 (10)	0.0487 (10)	0.0381 (9)	−0.0044 (8)	0.0122 (8)	−0.0032 (8)
C1	0.0348 (9)	0.0392 (9)	0.0334 (8)	0.0006 (7)	0.0059 (7)	0.0002 (7)
C16	0.0346 (9)	0.0459 (10)	0.0425 (10)	−0.0002 (8)	0.0126 (8)	0.0001 (8)
C3	0.0342 (10)	0.0584 (11)	0.0356 (9)	0.0001 (8)	0.0044 (8)	0.0035 (8)
C11	0.0486 (11)	0.0430 (10)	0.0462 (10)	−0.0027 (9)	0.0157 (9)	−0.0042 (8)
C27	0.0354 (9)	0.0495 (10)	0.0372 (9)	0.0088 (8)	0.0077 (8)	0.0050 (8)
C15	0.0377 (10)	0.0432 (10)	0.0323 (9)	−0.0003 (8)	0.0088 (7)	−0.0010 (7)
C2	0.0380 (10)	0.0523 (11)	0.0384 (9)	−0.0018 (8)	0.0047 (8)	−0.0022 (8)
N2	0.0618 (13)	0.0776 (14)	0.0797 (15)	0.0213 (11)	0.0359 (12)	0.0415 (12)
C8	0.0364 (10)	0.0525 (11)	0.0400 (10)	0.0029 (8)	0.0103 (8)	0.0088 (8)
C23	0.0352 (10)	0.0522 (11)	0.0487 (11)	0.0043 (8)	0.0106 (8)	0.0113 (9)
C24	0.0451 (11)	0.0619 (13)	0.0548 (12)	0.0168 (10)	0.0229 (10)	0.0215 (10)
C26	0.0589 (13)	0.0615 (13)	0.0362 (10)	0.0213 (11)	0.0078 (9)	0.0017 (9)
O3	0.1025 (15)	0.1061 (15)	0.0891 (13)	0.0394 (12)	0.0579 (12)	0.0579 (11)
C25	0.0669 (14)	0.0724 (14)	0.0402 (11)	0.0292 (12)	0.0234 (10)	0.0174 (10)
C4	0.0450 (12)	0.0729 (14)	0.0485 (11)	−0.0028 (11)	−0.0022 (9)	−0.0050 (10)
C28	0.0593 (13)	0.0470 (11)	0.0654 (13)	0.0083 (10)	0.0145 (11)	0.0086 (10)
C29	0.0500 (12)	0.0595 (12)	0.0526 (12)	−0.0089 (10)	0.0061 (10)	−0.0104 (10)
C21	0.0457 (12)	0.0549 (12)	0.0732 (14)	0.0015 (10)	0.0132 (11)	−0.0130 (11)
C7	0.0457 (12)	0.0596 (13)	0.0654 (14)	0.0090 (10)	0.0059 (11)	0.0089 (11)
C19	0.0393 (12)	0.0793 (16)	0.0898 (17)	−0.0087 (11)	0.0158 (12)	−0.0193 (14)
C18	0.0400 (11)	0.0662 (14)	0.0813 (16)	0.0054 (10)	0.0168 (11)	−0.0123 (12)
O4	0.1032 (17)	0.1027 (16)	0.1141 (18)	−0.0339 (14)	0.0253 (14)	0.0357 (14)
C17	0.0417 (11)	0.0503 (12)	0.0751 (15)	0.0034 (9)	0.0140 (10)	0.0007 (10)
C5	0.0402 (12)	0.0983 (19)	0.0620 (14)	0.0048 (12)	−0.0087 (11)	0.0015 (14)
C6	0.0434 (13)	0.0856 (18)	0.0755 (16)	0.0171 (12)	−0.0030 (12)	0.0097 (14)
C20	0.0544 (14)	0.0601 (14)	0.108 (2)	−0.0151 (11)	0.0187 (14)	−0.0168 (14)

### Geometric parameters (Å, °)

**Table:**

O2—C27	1.383 (2)	C23—C24	1.382 (3)
O2—C13	1.405 (2)	C23—H23	0.9300
N1—C2	1.386 (2)	C24—C25	1.373 (3)
N1—C14	1.415 (2)	C26—C25	1.379 (3)
N1—C1	1.472 (2)	C26—H26	0.9300
O5—C15	1.219 (2)	C25—H25	0.9300
C13—C12	1.377 (2)	C4—C5	1.373 (3)
C13—C14	1.412 (2)	C4—H4	0.9300
C14—C9	1.421 (2)	C28—H28A	0.9600
N3—C15	1.337 (2)	C28—H28B	0.9600
N3—C16	1.466 (2)	C28—H28C	0.9600
N3—H3	0.8600	C29—H29A	0.9600
C10—C11	1.374 (3)	C29—H29B	0.9600
C10—C9	1.419 (3)	C29—H29C	0.9600
C10—C28	1.510 (3)	C29—H29D	0.9600
C9—C8	1.466 (3)	C29—H29E	0.9600
C22—C27	1.378 (3)	C29—H29F	0.9600
C22—C23	1.381 (3)	C21—C20	1.519 (3)
C22—C1	1.503 (2)	C21—H21A	0.9700
O1—C2	1.223 (2)	C21—H21B	0.9700
C12—C11	1.389 (3)	C7—C6	1.375 (3)
C12—C29	1.505 (3)	C7—H7	0.9300
C1—C15	1.540 (2)	C19—C18	1.508 (3)
C1—H1	0.9800	C19—C20	1.521 (4)
C16—C21	1.514 (3)	C19—H19A	0.9700
C16—C17	1.517 (3)	C19—H19B	0.9700
C16—H16	0.9800	C18—C17	1.524 (3)
C3—C4	1.400 (3)	C18—H18A	0.9700
C3—C8	1.401 (3)	C18—H18B	0.9700
C3—C2	1.461 (3)	C17—H17A	0.9700
C11—H11	0.9300	C17—H17B	0.9700
C27—C26	1.384 (3)	C5—C6	1.378 (4)
N2—O4	1.217 (3)	C5—H5	0.9300
N2—O3	1.221 (3)	C6—H6	0.9300
N2—C24	1.474 (3)	C20—H20A	0.9700
C8—C7	1.404 (3)	C20—H20B	0.9700
			
C27—O2—C13	115.38 (13)	C5—C4—C3	119.9 (2)
C2—N1—C14	123.40 (15)	C5—C4—H4	120.0
C2—N1—C1	114.14 (14)	C3—C4—H4	120.0
C14—N1—C1	122.12 (13)	C10—C28—H28A	109.5
C12—C13—O2	114.03 (15)	C10—C28—H28B	109.5
C12—C13—C14	122.24 (16)	H28A—C28—H28B	109.5
O2—C13—C14	123.68 (15)	C10—C28—H28C	109.5
C13—C14—N1	122.13 (15)	H28A—C28—H28C	109.5
C13—C14—C9	118.32 (16)	H28B—C28—H28C	109.5
N1—C14—C9	119.54 (15)	C12—C29—H29A	109.5
C15—N3—C16	121.20 (15)	C12—C29—H29B	109.5
C15—N3—H3	119.4	H29A—C29—H29B	109.5
C16—N3—H3	119.4	C12—C29—H29C	109.5
C11—C10—C9	119.59 (17)	H29A—C29—H29C	109.5
C11—C10—C28	116.01 (18)	H29B—C29—H29C	109.5
C9—C10—C28	124.19 (18)	C12—C29—H29D	109.5
C14—C9—C10	118.47 (16)	H29A—C29—H29D	141.1
C14—C9—C8	117.61 (16)	H29B—C29—H29D	56.3
C10—C9—C8	123.91 (16)	H29C—C29—H29D	56.3
C27—C22—C23	119.37 (17)	C12—C29—H29E	109.5
C27—C22—C1	116.93 (16)	H29A—C29—H29E	56.3
C23—C22—C1	123.55 (17)	H29B—C29—H29E	141.1
C13—C12—C11	117.88 (17)	H29C—C29—H29E	56.3
C13—C12—C29	121.21 (17)	H29D—C29—H29E	109.5
C11—C12—C29	120.83 (17)	C12—C29—H29F	109.5
N1—C1—C22	109.29 (14)	H29A—C29—H29F	56.3
N1—C1—C15	110.02 (14)	H29B—C29—H29F	56.3
C22—C1—C15	117.11 (14)	H29C—C29—H29F	141.1
N1—C1—H1	106.6	H29D—C29—H29F	109.5
C22—C1—H1	106.6	H29E—C29—H29F	109.5
C15—C1—H1	106.6	C16—C21—C20	110.38 (18)
N3—C16—C21	110.99 (15)	C16—C21—H21A	109.6
N3—C16—C17	110.60 (15)	C20—C21—H21A	109.6
C21—C16—C17	110.42 (17)	C16—C21—H21B	109.6
N3—C16—H16	108.2	C20—C21—H21B	109.6
C21—C16—H16	108.2	H21A—C21—H21B	108.1
C17—C16—H16	108.2	C6—C7—C8	121.4 (2)
C4—C3—C8	121.73 (18)	C6—C7—H7	119.3
C4—C3—C2	116.84 (18)	C8—C7—H7	119.3
C8—C3—C2	121.33 (16)	C18—C19—C20	110.2 (2)
C10—C11—C12	122.71 (18)	C18—C19—H19A	109.6
C10—C11—H11	118.6	C20—C19—H19A	109.6
C12—C11—H11	118.6	C18—C19—H19B	109.6
C22—C27—O2	116.68 (15)	C20—C19—H19B	109.6
C22—C27—C26	122.35 (19)	H19A—C19—H19B	108.1
O2—C27—C26	120.94 (18)	C19—C18—C17	111.34 (18)
O5—C15—N3	123.95 (17)	C19—C18—H18A	109.4
O5—C15—C1	119.73 (16)	C17—C18—H18A	109.4
N3—C15—C1	116.14 (15)	C19—C18—H18B	109.4
O1—C2—N1	120.96 (17)	C17—C18—H18B	109.4
O1—C2—C3	122.76 (17)	H18A—C18—H18B	108.0
N1—C2—C3	116.18 (16)	C16—C17—C18	110.87 (18)
O4—N2—O3	124.5 (2)	C16—C17—H17A	109.5
O4—N2—C24	118.7 (2)	C18—C17—H17A	109.5
O3—N2—C24	116.8 (3)	C16—C17—H17B	109.5
C3—C8—C7	116.36 (18)	C18—C17—H17B	109.5
C3—C8—C9	119.33 (16)	H17A—C17—H17B	108.1
C7—C8—C9	124.15 (18)	C4—C5—C6	119.3 (2)
C24—C23—C22	118.1 (2)	C4—C5—H5	120.4
C24—C23—H23	121.0	C6—C5—H5	120.4
C22—C23—H23	121.0	C7—C6—C5	121.2 (2)
C25—C24—C23	122.5 (2)	C7—C6—H6	119.4
C25—C24—N2	119.3 (2)	C5—C6—H6	119.4
C23—C24—N2	118.2 (2)	C19—C20—C21	111.0 (2)
C25—C26—C27	118.1 (2)	C19—C20—H20A	109.4
C25—C26—H26	120.9	C21—C20—H20A	109.4
C27—C26—H26	120.9	C19—C20—H20B	109.4
C24—C25—C26	119.56 (18)	C21—C20—H20B	109.4
C24—C25—H25	120.2	H20A—C20—H20B	108.0
C26—C25—H25	120.2		
			
C27—O2—C13—C12	129.21 (16)	N1—C1—C15—N3	161.90 (15)
C27—O2—C13—C14	−53.3 (2)	C22—C1—C15—N3	36.3 (2)
C12—C13—C14—N1	171.12 (16)	C14—N1—C2—O1	169.55 (18)
O2—C13—C14—N1	−6.2 (3)	C1—N1—C2—O1	−3.9 (3)
C12—C13—C14—C9	−7.3 (3)	C14—N1—C2—C3	−13.8 (2)
O2—C13—C14—C9	175.38 (15)	C1—N1—C2—C3	172.76 (15)
C2—N1—C14—C13	−176.13 (16)	C4—C3—C2—O1	11.5 (3)
C1—N1—C14—C13	−3.2 (2)	C8—C3—C2—O1	−172.22 (19)
C2—N1—C14—C9	2.3 (2)	C4—C3—C2—N1	−165.04 (17)
C1—N1—C14—C9	175.19 (15)	C8—C3—C2—N1	11.2 (3)
C13—C14—C9—C10	10.7 (2)	C4—C3—C8—C7	3.0 (3)
N1—C14—C9—C10	−167.78 (15)	C2—C3—C8—C7	−173.10 (18)
C13—C14—C9—C8	−169.55 (15)	C4—C3—C8—C9	178.60 (18)
N1—C14—C9—C8	12.0 (2)	C2—C3—C8—C9	2.5 (3)
C11—C10—C9—C14	−7.5 (3)	C14—C9—C8—C3	−14.1 (2)
C28—C10—C9—C14	167.06 (17)	C10—C9—C8—C3	165.58 (17)
C11—C10—C9—C8	172.82 (17)	C14—C9—C8—C7	161.12 (18)
C28—C10—C9—C8	−12.7 (3)	C10—C9—C8—C7	−19.1 (3)
O2—C13—C12—C11	177.86 (15)	C27—C22—C23—C24	−0.3 (3)
C14—C13—C12—C11	0.3 (3)	C1—C22—C23—C24	−175.65 (17)
O2—C13—C12—C29	1.1 (2)	C22—C23—C24—C25	−0.6 (3)
C14—C13—C12—C29	−176.40 (17)	C22—C23—C24—N2	179.31 (17)
C2—N1—C1—C22	−124.19 (16)	O4—N2—C24—C25	170.6 (2)
C14—N1—C1—C22	62.3 (2)	O3—N2—C24—C25	−11.2 (3)
C2—N1—C1—C15	105.92 (17)	O4—N2—C24—C23	−9.4 (3)
C14—N1—C1—C15	−67.58 (19)	O3—N2—C24—C23	168.84 (19)
C27—C22—C1—N1	−69.71 (19)	C22—C27—C26—C25	−1.8 (3)
C23—C22—C1—N1	105.78 (19)	O2—C27—C26—C25	−179.40 (17)
C27—C22—C1—C15	56.2 (2)	C23—C24—C25—C26	0.3 (3)
C23—C22—C1—C15	−128.31 (18)	N2—C24—C25—C26	−179.63 (18)
C15—N3—C16—C21	70.1 (2)	C27—C26—C25—C24	0.9 (3)
C15—N3—C16—C17	−167.00 (17)	C8—C3—C4—C5	−0.6 (3)
C9—C10—C11—C12	0.4 (3)	C2—C3—C4—C5	175.6 (2)
C28—C10—C11—C12	−174.57 (18)	N3—C16—C21—C20	−179.58 (19)
C13—C12—C11—C10	3.3 (3)	C17—C16—C21—C20	57.4 (2)
C29—C12—C11—C10	180.00 (18)	C3—C8—C7—C6	−3.3 (3)
C23—C22—C27—O2	179.20 (15)	C9—C8—C7—C6	−178.7 (2)
C1—C22—C27—O2	−5.1 (2)	C20—C19—C18—C17	−56.0 (3)
C23—C22—C27—C26	1.5 (3)	N3—C16—C17—C18	−179.81 (18)
C1—C22—C27—C26	177.21 (17)	C21—C16—C17—C18	−56.6 (2)
C13—O2—C27—C22	75.73 (19)	C19—C18—C17—C16	56.3 (3)
C13—O2—C27—C26	−106.56 (19)	C3—C4—C5—C6	−1.5 (4)
C16—N3—C15—O5	12.4 (3)	C8—C7—C6—C5	1.4 (4)
C16—N3—C15—C1	−172.54 (15)	C4—C5—C6—C7	1.1 (4)
N1—C1—C15—O5	−22.8 (2)	C18—C19—C20—C21	57.0 (3)
C22—C1—C15—O5	−148.36 (17)	C16—C21—C20—C19	−57.9 (3)

### Hydrogen-bond geometry (Å, °)

**Table:**

*D*—H···*A*	*D*—H	H···*A*	*D*···*A*	*D*—H···*A*
N3—H3···O3^i^	0.86	2.29	3.046 (2)	147

Symmetry codes: (i) −*x*+1, −*y*, −*z*+2.
